# Ophthalmology

**Published:** 1892-09

**Authors:** F. R. Cross


### OPHTHALMOLOGY.

At this season of the year, when the continuous routine of medical work is for a time suspended, and when doctors, in common with the public, are all bent upon vacation and a change of air, we cannot but recognise the general ability with which the great railway and navigation companies arrange their traffic, and the satisfactory and courteous manner in which their numerous officials carry out the extra strain and labour thrust upon them at holiday time. Despite the addition of special excursions to the overburthened general service, punctuality is fairly maintained, and accidents, in this country at any rate, are of very exceptional occurrence.

For practical purposes, the organisation for travel, both by sea and land, seems to be as perfect as can be fairly demanded; but an examination into detail from a scientific standpoint indicates that one of the most important links in the chain of safety still goes untested, while its strength, unproven, is taken almost simply on faith. However thoroughly the machinery of the engines may be tried, however carefully the roads may be watched and mended, and however well the rules for transit may be elaborated and fulfilled, a factor of not less importance to safety than any other in this connection exists wherever the eyesight of employés is of primary importance, as in the case of engine-drivers and signalmen.

Though it may not be confessed or understood, many accidents have resulted through imperfect eyesight, whether for coloured signals, or for distant or close objects, or for range. The possible existence of imperfect eyesight and the necessity for its detection in those who apply for positions in railway employ, is admitted by the companies, who submit all candidates to an examination of some kind or other. Dr. George Mackay of Edinburgh showed in a paper published in the *British Medical Journal*, August 29th, 1891, that the tests used on the Scottish railways are quite inadequate, either for testing the colour sense or the general power of sight. Mr. Beaumont, of Bath, in the same journal, shows from his own experience how incompetent, for what is required of them, is the sight of many officials. He insists on the importance of timely re-examination, it being obvious that all kinds of defects may supervene in course of years. Mr. McHardy, in the same number, mentions the case of a signalman "who must have been for many years too blind to distinguish a man from a woman at ten yards." This man had, only a short time before, been re-examined and passed by the railway surgeon with the "practical" tests in vogue.

As the outcome of these papers and the discussion which followed them in the section of Ophthalmology at the annual meeting of the British Medical Association, at Bournemouth, last year, "A committee to promote the efficient control of railway servants' eyesight" was appointed by the Council of the Association. The Report of this committee has now been published; it deals with the whole subject in a masterly way. Copies can be obtained at the Office of the Association, 429 Strand, London. It has been sent to every passenger railway manager in the United Kingdom. The Report shows that in 1877 Professor Holmgren's work on "Colour Blindness and its relation to Railroads and the Marine," was published in Upsala. The method of testing for colour with skeins of wool (which had been employed by Wilson of Edinburgh, who seems to have been the pioneer in this question some twenty years previously) was elaborated. In the same year, Donders of Utrecht published papers "On the Quantitative Estimation of the Colour Sense," and a "Report on the Eyesight of the Employés on the Dutch State Railways." Our Board of Trade, in 1877, also issued regulations for testing the colour sense of candidates for higher posts in the mercantile marine, but they dealt solely with the question of colour sense; the capacity for seeing distant objects was left unconsidered.

At the International Medical Congress held at Amsterdam in 1879, Donders brought forward a series of suggestions for testing the eyesight of railway servants; and at the Congress held in London in 1881 resolutions were adopted as to "Tests of Sight suitable to be enforced in the case of Signallers and Look-out Men and other persons by Land or Sea;" together with "Suggestions as to International Arrangements for a Uniform System of Maritime, Coast, and Harbour Signalling, with a view to the Safety of Life and Property." It was recommended that for admission to any railway service there should be required: A healthy condition as regards habitual congestion or irritation of the eyes and eyelids; for each eye complete field of vision; total absence of cataract or any other progressive disease; in one eye normal acuity and refraction, colour sense $\frac{3}{5}$, and in the other eye at least $\frac{1}{2}$ vision both as regards acuity and colour sense. Drivers or stokers, however, should possess normal acuity and refraction, with colour sense at least $\frac{4}{5}$, etc.

To ensure the perfection of the system, and its uniformity, a central medical authority was considered requisite. He should propose the examiners, and be responsible for their fitness. They should be men of ascertained competency, and, as far as practicable, qualified as medical specialists.

In Holland the arrangements for the control of railway servants' eyesight are very perfect. Prof. Snellen is the chief controlling authority, and ophthalmic surgeons in different towns of Holland act as examiners for certain sections of the lines.

In Sweden, France, Italy, and the State of Alabama, careful testing is adopted in the case of every candidate: it is invariably entrusted to medical men; in Holland and Italy to ophthalmic surgeons only.

In England, however, only four of the railway companies, and in Ireland two, employ medical men to examine the eyesight of *all* applicants. Most other companies simply employ men selected from the employés to test the visual fitness of firemen and engine-drivers. There is no uniform system: a man refused by one company might find no difficulty in obtaining occupation in another. The present system is eminently unsatisfactory, in the interests of the employés, as well as in those of the companies, and of the travelling public.

Two cases in my experience indicate possibilities under the existing system. A signalman near Pill was found to be completely colour-blind, and was at once personally reported to the manager, on the understanding that he should, without loss of pay, be given other work not requiring colour vision. A driver of an express train suddenly found himself blinded by a fragment being blown into one conjunctival sac; he, for the first time, discovered that his other eye was quite useless, and the train had to be left under the control of the intelligence and power of sight of the stoker. The faulty eye proved only to be highly myopic.

It is no trustworthy test for colour vision to ask a candidate to name a coloured flag, or dot, or lamp, as is usually done by the railway authorities. However difficult it has hitherto been to impress this on the lay mind, the Report recently issued by the Royal Society's Committee in answer to the Board of Trade, "What is the Best Test for Colour Blindness?" will probably be accepted as a conclusive answer. "The Committee is of opinion that tests which involve the naming of colour should be avoided in deciding the question of colour-blindness. . . . After practical trials, and also from theoretical considerations, the Committee are of opinion that the simplest efficient test is the wool test of Holmgren, applied in the form which Holmgren himself recommends." The Committee consider that the colour testing for the Royal Navy is most efficient, but they consider that the tests which the Board of Trade have officially adopted, and the methods of applying them, are open to very grave objections. Concerning the railway companies, "the evidence shows that few have any adequate system of testing; nearly all the methods employed are defective."

A thorough examination by competent examiners is a very simple matter as no costly reform is required. A properly-conducted examination for colour under the method of Holmgren (supplemented by a search for scotomata, such as may result from the excessive use of tobacco), and for form under the method of Snellen, would enable the companies to make perfectly trustworthy selection of men for special purposes. Each eye must be separately tested for colour as well as form, and periodical re-examination is necessary.

Now that the attention of the Board of Trade and of the railway authorities has been again called to this subject, it is to be hoped that voluntary reform will be undertaken without delay, and before the public are obliged to demand the interference of the State.

F. R. Cross.

